# Differentially Expressed MicroRNAs in Radioresistant and Radiosensitive Atypical Meningioma: A Clinical Study in Chinese Patients

**DOI:** 10.3389/fonc.2020.00501

**Published:** 2020-04-30

**Authors:** Xiaokang Zhang, Guobin Zhang, Huawei Huang, Haoyi Li, Song Lin, Yonggang Wang

**Affiliations:** ^1^Department of Neurosurgery, Beijing Tiantan Hospital, Capital Medical University, Beijing, China; ^2^Department of Critical Care Medicine, Beijing Tiantan Hospital, Capital Medical University, Beijing, China

**Keywords:** atypical meningioma, radioresistance, radiosensitivity, microRNA, adjuvant radiotherapy

## Abstract

**Background:** For atypical meningiomas (AMs), the combination of gross total resection (GTR) and adjuvant radiotherapy (ART) is still a controversial therapeutic strategy to improve prognosis. This study analyzed the factors influencing the prognosis on AM patients treated with GTR + ART by investigating both clinical characteristics and the change in microRNA (miRNA) expression.

**Materials and Methods:** Adult AM patients who were admitted to the Tiantan hospital from 2008 to 2015 and underwent GTR + ART were included. Patients who suffered recurrence within 3 years after operation were considered radioresistant, while the others were considered radiosensitive. Clinical characterizations were compared between these two groups. The microRNA (miRNA) expression was detected via miRNA microarray in 10 patients, five from the radiosensitive group and from the radioresistant group.

**Results:** A total of 55 cases were included in this study. No significant difference was found in the clinical characteristics (gender, age, tumor location, tumor size, peritumoral brain edema, and Ki-67 index) between radiosensitive and radioresistant patients. We found seven significantly upregulated miRNAs (miR-4286, miR-4695-5p, miR-6732-5p, miR-6855-5p, miR-7977, miR-6765-3p, miR-6787-5p) and seven significantly downregulated miRNAs (miR-1275, miR-30c-1-3p, miR-4449, miR-4539, miR-4684-3p, miR-6129, miR-6891-5p) in patients resistant to radiotherapy. The differentially expressed miRNAs were enriched mostly in the fatty acid metabolic pathways (hsa00061, hsa01212) and transforming growth factor beta signaling pathway (hsa04350).

**Conclusion:** For AM patients treated with GTR + ART, the changes in miRNA expression discovered in this study may be a potential predictor of individual sensitivity to adjuvant radiotherapy. Further research is needed regarding the predictive power and mechanism by which these miRNAs influence prognosis.

## Introduction

Meningioma, which has an incidence of 6–7 in 100,000 people, has become the most common primary brain tumor, accounting for 36.3% of all primary central nervous system tumors (1). According to the World Health Organization (WHO) criteria, meningioma is currently classified as grade I, II or atypical, and III or anaplastic (2), representing 80, 5–34, and 1–3% (3) of all meningiomas, respectively. Although most meningiomas are benign, the non-benign meningiomas are associated with poor prognosis, including aggressive behavior, and early tumor recurrence or progression (4). As such, for high-grade meningioma, adjuvant radiotherapy (ART) is an important method to control tumor recurrence after surgical resection.

However, due to the controversial results obtained between different studies, whether the ART is beneficial for the treatment of atypical meningioma (AM) patients, especially for those who underwent gross total resection (GTR, Simpson I–II), remains unclear (4–11). Presently, for AM patients, the decision to perform ART after GTR is often based on the experience of the clinician. To our knowledge, the only completed prospective study regarding ART after GTR in AM is European Organization for Research and Treatment of Cancer (EORTC) 22042-26042, which showed that the 3-year progression-free survival (PFS) for AM patients undergoing complete resection (Simpson I–III) followed by treatment with high-dose (60 Gy) radiotherapy is over 70% (12). However, this study provides information regarding the dose and administration of ART but does not address whether ART is necessary after GTR.

During the past two decades, the WHO classification system was significantly revised in 2000 and updated in 2007 and then in 2016 (2). With these changes, the criterions for AM have been “enlarged” (by adding brain invasion as a criterion for the diagnosis of AM), and hence, the incidence of AM has tended to increase throughout time (13). Biological heterogeneity complicates this issue, as certain AM patients may be inherently more insensitive to a given dose of radiation. Failure to control a tumor with a seemingly curative dose would suggest that the tumor is “radioresistant,” whereas a “radiosensitive” tumor would be controlled via radiotherapy. When considering radiation toxicity and the lack of consensus among neurosurgeons and meningioma researchers, the decision for ART in AM patients after GTR should be individualized. Thus, predictive strategies to determine the radiosensitivity of AM patients are required to facilitate the future delivery of personalized radiotherapy. MicroRNAs (miRNAs) are a type of small non-coding RNA (containing about 22 nucleotides), which plays a role in RNA silencing and posttranscriptional regulation of gene expression. Moreover, miRNAs are getting increasing attention as potential markers of tumor radiosensitivity and have shown potential in several other malignancies (14–18).

In our institution, after surgical resection, we routinely recommend patients with AM to consult a radiotherapy specialist for further treatment. Interestingly, we found that even for those AM patients who underwent GTR and ART, there were still some differences in prognosis. Therefore, in order to investigate the correlation between radiotherapy sensitivity and miRNA expression, we conducted an extensive miRNA profiling study on tissue samples from postoperative radiotherapy-sensitive and radiotherapy-resistant AM patients who underwent GTR and ART in a single institution and searched for unique miRNA expression signatures that could distinguish radiotherapy-sensitive patients from radiotherapy-resistant patients.

## Materials and Methods

### Ethics Statement

All patients enrolled in the study signed an informed consent form for the current study, and the clinical study was approved by the Medical Ethics Committee of the Capital Medical University.

### Patients and Tumor Specimens

Patients diagnosed with AM from 2008 to 2015 were initially identified through the database of our Neurosurgery department at the Beijing Tiantan Hospital. The clinical history of the patients was gathered retrospectively by chart review. Fifty-nine AM patients who underwent gross total resection were identified and selected for further analysis. The operation notes and postoperative magnetic resonance images (MRIs) were reviewed to confirm the extent of the resection. Simpson I (macroscopically complete tumor resection with removal of affected dura and underlying bone)–II (macroscopically complete tumor resection with coagulation of affected dura only) was defined as GTR (19). The pathological reports were reviewed, and all pathological diagnoses were examined and graded independently by two neuropathologists (who were blind to tumor genotypes), according to the 2016 World Health Organization (WHO) Classification of Tumors of the Central Nervous System (2). The external-beam radiation was delivered by conventional fractionation up to a total dose of 50–60 Gy. The exclusion criteria included the age <18 years old (one case), having other intracranial or systematic malignant tumors before/concurrent (two cases), extracranial tumor location, and loss to follow-up (one case). Therefore, a total of 55 cases were included in this study.

In recent studies, recurrence-free survival in 3 years has been a critical prognostic indicator to estimate the efficiency of radiotherapy for atypical meningioma. Since a prospective study confirmed GTR + ART could make PFS in 3 years >70% (12), in this study, patients who suffered tumor recurrence within 3 years (36 months) after GTR + ART were defined as the radioresistant group, while the others were defined as the radiosensitive group. Patient characteristics, including gender, age (≤ 60 vs. >60), tumor location, preoperative tumor size, Ki-67 index, and peritumoral brain edema (PTBE), were compared between these two groups. According to their location, tumors were divided into five categories: convexity (including frontal, temporal, parietal, and occipital), falx/parasagittal, cranial base (e.g., olfactory groove, sphenoid ridge, petroclival region, tuberculum sellae, etc.), lateral ventricle trigone area, and posterior fossa (19). Preoperative MRIs were reviewed to measure tumor size (the longest axis rounded to the nearest millimeter, divided at 4.5 cm) and PTBE. Recurrence-free survival (RFS) was measured from the date of the surgery to the date of death/last follow-up/progression based on the first radiographic documentation, whichever occurred first.

For every patient, immediately after surgery, tumor samples were fixed with formalin and embedded in paraffin blocks. A random selection was made to obtain 10 tumor samples for further miRNA microarray test, with five samples from the radioresistant group and five from the radiosensitive group.

### MicroRNA Microarray

miRNAs were extracted from formalin-fixed and paraffin-embedded tissues using the miRNeasy Mini Kit (QIAGEN) according to the manufacturer's instructions. The concentration and purity of the RNA were measured using the NanoDrop 1000 spectrophotometer (Thermo Fisher Scientific). The quality of the total RNA was accessed using the Agilent 2100 Bioanalyzer (Agilent Technologies, Santa Clara, CA, USA). A total of 200 ng of small RNAs were labeled using the FlashTag biotin-HSR RNA labeling kit (Genisphere). First, poly(A) tailing was carried out at 37°C for 15 min in a volume of 15 μl of reaction mixture containing the reaction buffer, MnCl_2_, ATP, and poly(A) polymerase. Then, the Genisphere biotin complex was ligated at room temperature for 30 min by adding the FlashTag Ligation Mix Biotin and T4 DNA Ligase into the 15-μl reaction mix. The Stop Solution was then added to stop the reaction.

Subsequently, the microRNA cocktails were hybridized and analyzed on microRNAs microarrays version 2 or 3 (Affymetrix). Labeled RNAs were hybridized on GeneChip microarrays, washed, stained, and then scanned using the miRNA-2.0 library for microRNA microarrays version 2 and the miRNA-3.0 library for microRNA microarrays version 3, according to Affymetrix's specifications.

### Statistical Analysis

Analyses of clinical data were performed using the SPSS software (release version 21; IBM Corp., Armonk, NY, USA). The tumor size and Ki67 index between groups were compared by independent samples *t*-test. Pearson's chi-square test was used to compare gender, tumor location, and PTBE between groups. Death by the last follow-up was compared using Fisher's exact test. The median RFS of both groups was calculated using the Kaplan–Meier method and compared by log-rank tests. A *p* < 0.05 was regarded as statistically significant.

For microRNA data, the signal intensity was loaded into the Rosetta Resolver System® (Rosetta Biosoftware, USA) for data preprocessing and application of the 75th percentile centering normalization. Simultaneously, the errors of the sample were estimated using the error-weighted approach. Both the fold change and *p*-value for pairwise sample comparisons were calculated to evaluate differentially expressed genes. MiRNAs with a fold change of ≥ 2 or ≤ −2 and a *p* < 0.05 were considered as differentially expressed. Hierarchical clustering was performed using iDEP (20). Significantly upregulated and downregulated miRNAs were selected for pathway analysis using the DNA Intelligent Analysis (DIANA)-miRPath v3.0 software, according to a previously published protocol (21). Briefly, this software is able to link miRNAs to experimentally validated target genes from Tarbase, v7.0, and identify the putative targeted molecular pathways in the Kyoto Encyclopedia of Genes and Genomes (KEGG) (22). The “pathways union” option of the miRPath software was selected, and *p*-values were obtained using Fisher's exact test.

## Results

### Patient Characteristics Analysis

Fifty-five cases of AM were included in this study. For all cases, the last follow-up was in December 2018, with a median follow-up time of 57 months (range, 37–127). A summary of patient characteristics is shown in Table 1. A total of 43 patients fulfilled the criteria for the radiosensitive group, and 12 patients were included in the radioresistant group. The radioresistant group consists of 12 cases with a median age of 52 and with most patients younger than 60 years (72%). Similarly, the radiosensitive group consists of 43 cases with a median age of 52 and 83.7% of patients younger than 60 years. Consequently, we failed to find a significant difference regarding age (*p* = 0.673, Fisher's exact test) and gender (*p* = 0.192, Pearson's chi-square test) between the radiosensitive and radioresistant group. There was also no significant difference regarding tumor location, tumor size, and the Ki-67 index between the radiosensitive and radioresistant group. In this study, most tumors were located in the supratentorial area (7 of the radioresistant and 27 of the radiosensitive, *p* = 0.779, Pearson's chi-square test). In the radioresistant group, tumors were most commonly located in the brain convexity and cranial base, respectively, in three patients (25%), followed by falx/parasagittal (16.7%), the lateral ventricle trigone area (16.7%), and the posterior fossa (16.7%). The tumor location of the radioresistant group was not significantly different from the radiosensitive group, which were located in convexity (34.9%), followed by falx/parasagittal (23.2%), the cranial base (18.6%), the posterior fossa (18.6%), and the lateral ventricle trigone area (4.7%). According to the preoperative MRI, median tumor size was 5.95 cm in the radioresistant group and 5.00 cm in the radiosensitive group (*p* = 0.265, independent samples *t*-test). Six patients from the radioresistant group and 11 patients from the radiosensitive group suffered PTBE, but there was no significant difference between these two groups (*p* = 0.177, Pearson's chi-square test). The mean Ki-67 index was 11.5% in the radioresistant group, which was not significantly different (*p* = 0.343, independent samples *t*-test) from that of the radiosensitive group (8.4%). Median RFS differed significantly between the two groups (*p* < 0.001, log-rank test), with 28.5 months in the radioresistant group and 58 months in the radiosensitive group.

**Table 1:** Clinical characteristics of the atypical meningioma patients with adjuvant radiotherapy after gross total resection.

**Characteristics**	**Radiosensitive** **(*n* = 43)**	**Radioresistant** **(*n* = 12)**	***p*-value**
Gender (male/female)	25/18	4/8	0.192
Median age at surgery	52	52	0.673
Tumor location			
Supratentorial (yes/no)	27/16	7/5	0.779
Convexity (%)	15 (34.9%)	3 (25.0%)	0.519
Falx/parasagittal (%)	10 (23.2%)	2 (16.7%)	0.625
Cranial base (%)	8 (18.6%)	3 (25.0%)	0.624
Lateral ventricle trigone area (%)	2 (4.7%)	2 (16.7%)	0.204
Posterior fossa (%)	8 (18.6%)	2 (16.7%)	0.878
Median tumor size (cm)	5.00	5.95	0.265
PTBE (with/without)	11/32	6/6	0.158
Mean Ki 67 index	8.4%	11.5%	0.343
Death by the last follow-up	2	1	0.117
Median RFS (months)	58	28.5	<0.001
Median follow-up (months)	57 (36–127)		

*PTBE, peritumoral brain edema; RFS, recurrence-free survival*.

### MicroRNA Characterization

**Figure 1:**
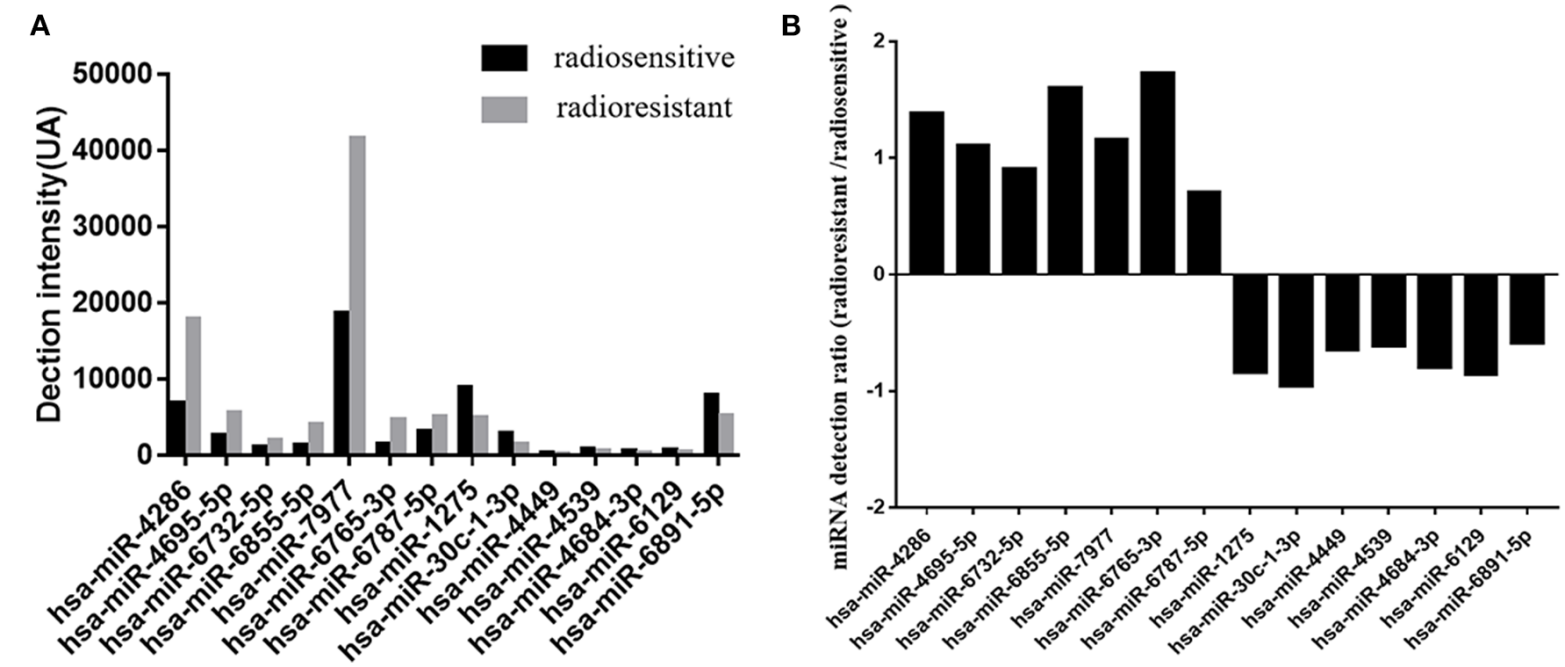
Differential expression microRNAs (miRNAs) in the radiosensitive and radioresistant groups of atypical meningioma with gross total resection plus adjuvant radiotherapy (GTR + ART). **(A)** Detection levels on Affymetrix microarrays of the microRNAs in radioresistant group (*gray*) and radiosensitive group (*black*). Detection intensities correspond to the measured values minus the threshold value. **(B)** The ratios of the measured intensities of microRNAs detected in radioresistant group vs. the intensities in radiosensitive group. The ratios are shown on a log_2_ scale.

**Figure 2:**
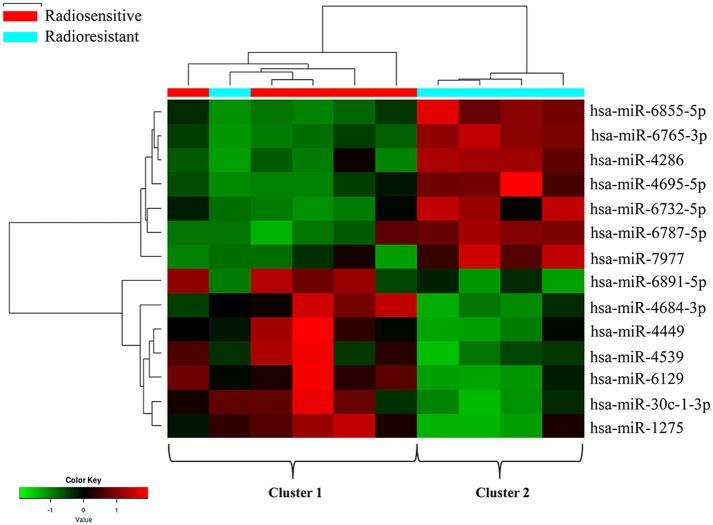
Clustering analysis of the radioresistant and radiosensitive groups using differentially expressed miRNAs. The columns represent the cases, and the lines represent the miRNAs. Red and green indicate high and low expression levels, respectively. Cluster 1: five radiosensitive and one radioresistant; cluster 2: radioresistant.

In this study, tumor samples from 10 patients were selected for miRNA microarray. Five of them suffered tumor recurrence <3 years (36 months) after total resection, which was considered as radioresistant, while the others who did not exhibit tumor recurrence during the follow-up time (>36 months) were considered as radiosensitive. The clinical characteristics of these 10 patients are shown in Table 2. Between the radiosensitive and radioresistant group, there was no significant difference in gender, age, tumor location, tumor size, PTBE, and Ki67 index, and no patients suffered severe disease in history. A comparison between the miRNA profiles of the radioresistant and radiosensitive group AM samples revealed 1,466 common miRNAs. We observed 14 significant differentially expressed miRNAs between the radiosensitive and radioresistant cases (Figure 1A). Of these, seven were upregulated (miR-4286, miR-4695-5p, miR-6732-5p, miR-6855-5p, miR-7977, miR-6765-3p, miR-6787-5p), while seven were downregulated (miR-1275, miR-30c-1-3p, miR-4449, miR-4539, miR-4684-3p, miR-6129, miR-6891-5p) in the radioresistant cases (Figure 1B). Unsupervised hierarchical clustering was performed using iDEP (20). This led to the separation of all the cases into two main clusters, as shown in Figure 2. Cluster 1 included five out of six (83.3%) radiosensitive cases, while cluster 2 consisted of the radioresistant cases (four out of four, 100%). The DIANA-miRPath v.3 software (21) was used to explore the biological significance of the 14 miRNAs that were differentially expressed between the radioresistant and radiosensitive group. Three enriched pathways were revealed by this analysis (Table 3). According to the KEGG pathway maps, one pathway was the environmental information processing related pathway [transforming growth factor beta (TGF-β) signaling pathway, hsa04350], and the other two were related to metabolic system pathways (fatty acid biosynthesis, hsa00061; fatty acid metabolism, hsa01212).

**Table 2:** Clinical characteristics of patients whose tumor was detected by microRNA (miRNA) microarray.

**Patient ID**	**Gender**	**Age**	**Tumor location**	**Tumor size (cm)**	**PTBE**	**Ki 67 index (%)**	**Past medical history**
C1	Female	33	Convexity	4.5	Yes	1	No
C2	Female	53	Convexity	5.0	Yes	1	Endometrial polyp with resection
C3	Female	54	Posterior fossa	4.8	No	20	Ovarian cyst with resection
C4	Male	18	Cranial base	3.1	Yes	5	No
C5	Female	47	Lateral ventricle trigone area	4.9	No	3	Uterine fibroids
T1	Female	64	Convexity	4.4	Yes	15	Hypertension for 5 years
T2	Female	34	Lateral ventricle trigone area	5.9	No	5	No
T3	Male	24	Posterior fossa	2.7	No	1	No
T4	Female	44	Cranial base	4.1	Yes	30	Chronic superficial gastritis
T5	Male	58	Convexity	6.0	Yes	1	Inguinal hernia with repair
*p*-value	1.0	0.695	1.0	0.828	1.0	0.526	

*C1–C5, radiosensitive group; T2–T5, radioresistant group*.

**Table 3:** Results from the DIANA-miRPath v3.0 predictions of Kyoto Encyclopedia of Genes and Genomes (KEGG) pathways according to the differential expression microRNAs (miRNAs) between radioresistant and radiosensitive groups.

**KEGG pathway maps**	**Enriched pathway**	***p*-value**
Metabolism	Fatty acid biosynthesis (hsa00061)	<1 × 10–325
	Fatty acid metabolism (hsa01212)	4.21 × 10–06
Environmental information processing	TGF-beta signaling pathway (hsa04350)	0.040294

## Discussion

The use of ART for the treatment of AM after GTR has remained controversial. Maybe the single institution and relatively small study scale could be a partial reason for this contradiction. However, recently, two large-scale studies have not managed to reach a consensus. Wang C. et al. showed that ART is not associated with improved overall survival (OS) in patients who underwent GTR [adjusted hazard ratio (HR) = 1.093, *p* = 0.737] (23). However, Rydzewski N.R. et al. demonstrated that GTR in combination with ART was the most critical factor for improved survival (GTR plus ART, HR = 0.47; *p* = 0.002), even though GTR was associated with lower rates of adjuvant radiation usage based on the National Cancer Data Base (NCDB) (7). We speculate that this contradiction in findings could be due to some factors influencing tumor radiosensitivity, which were confounded in the above studies. In order to find interfering factors of the effect of ART after GTR in AM, our study focused on patients who underwent both GTR and ART and aimed to define factors associated with radiosensitivity.

As we all know, in order to make individual treatment decisions, clinicians should weigh and balance multiple factors at different levels. At the clinical level, this study failed to find a significant difference in the characteristics of patients with AM treated with GTR + ART. To explore the factors that exert more influence on these patients, especially to find those factors that contribute to radiosensitivity, we investigated differentially expressed miRNAs. Recent studies have revealed that differences in miRNA expression could influence radiosensitivity in a series of tumors, including, but not limited to, glioblastoma (GBM), breast cancer, lung cancer, melanoma, and cervical cancer (14–18). However, research regarding the role of miRNAs in AM radiosensitivity is scarce. In this study, we found 14 differentially expressed miRNA between radiosensitive and radioresistant AM patients. We identified seven upregulated miRNAs (miR-4286, miR-4695-5p, miR-6732-5p, miR-6855-5p, miR-7977, miR-6765-3p, miR-6787-5p) and seven downregulated miRNAs (miR-1275, miR-30c-1-3p, miR-4449, miR-4539, miR-4684-3p, miR-6129, miR-6891-5p) in the radioresistant group. According to this pattern of miRNA deregulation, these 10 samples could be divided into two clusters. Notably, the division pattern of these two clusters was nearly coincident with the radiosensitivity division. There was one special patient (subject T4 in Table 2) whose miRNA expression pattern was the same to that of the radiosensitive group was considered as radioresistant at clinical level due to the poor radiotherapy effect. Although the tumor located at sphenoid ridge and it is a relatively hard work to design and execute external-beam radiation in this region, the patient still chose to undertake radiotherapy at a local hospital and might experience an unsuccessful radiotherapy, which made the poor prognosis for this patient.

Among the 14 deregulated miRNAs, miR-7977, miR-4286, miR-1275, and miR-30c-1-3p have been previously reported to play a role in tumor malignancy. Horiguchi H. et al. found that miR-7977 was upregulated in acute myeloid leukemia and myelodysplastic syndrome and could reduce the expression of poly(rC) binding protein 1 to interfere with normal hematopoiesis. Moreover, miR-7977 was also reported to regulate the Hippo-YAP pathway, therefore inducing the upregulation of leukemia-supporting stroma growth (24, 25). MiR-4286 is another miRNA that was found to be upregulated in the radioresistant group in our study. In previous studies, its upregulation is also found to be associated with cell proliferation, migration, and invasion via targeting of PTEN and Runx3 (26, 27). As for the downregulated miRNAs identified in our work, miR-1275 was reported to inhibit cell migration and invasion in gastric cancer, while the downregulation of miR-1275 by H3K27me3 could mediate glial induction of GBM cells (28, 29). Furthermore, reduced expression of miR-30c-1-3p was also found in prostate cancer, while overexpression of miR-30c-1-3p was shown to inhibit the progression of prostate cancer (30).

Finally, in order to investigate the molecular pathways affected by the differentially expressed miRNAs between radiosensitive and radioresistant AM, we used the DIANA-miRPath software and found three enriched pathways. The two most significant pathways were fatty acid biosynthesis (hsa00061) and metabolism (hsa01212): fatty acid biosynthesis, biosynthesis, and TGF-β signaling pathways. These pathways have been verified relate to some common chronic disorders such as chronic inflammation, hypertension, and hyperlipidemia (31, 32); however, just as is shown in Table 2, these conditions were scarce in patients who undertook miRNA array test. As we all know, fatty acids are the principal constituent of cell membranes and essential components for the energy required for cancer growth. Changes in fatty acid synthesis and metabolism were identified in many different types of tumors and have been considered as a potential therapeutic target in cancer (33). Moreover, several studies have indicated that fatty acid regulation could influence the radiosensitivity of tumors such as prostate cancer and nasopharyngeal carcinoma (34, 35). However, research on fatty acid changes in meningioma is scarce, making it an area worthy of further exploration. The TGF-β signaling pathway is another pathway enriched in this study. There is accumulating evidence to show that the TGF-β signaling pathway is related to meningioma cell proliferation and contributes to the development and/or progression of higher-grade meningiomas (36–38). However, the relationship between the TGF-β signaling pathway and meningioma radiosensitivity remains unclear, thereby requiring further investigation.

The miRNA deregulation pattern discovered in this study could help to define radioresistant AM patients properly; this is important for follow-up treatment. On the one hand, AM patients who are radioresistant and vulnerable to radiation-induced injury could choose observation after GTR. On the other hand, these radioresistant patients are more worth trying radiosensitizer to improve the effect of radiotherapy. The use of miRNA as a kind of treatment method is quite far from clinical practice, but there are still a number of drugs to improve radiosensitivity. For now, several clinical trials about radiosensitizers have been done (39). Among these drugs, RRx-001(NCT02871843) and NVX-108 (NCT02189109) were two novel molecules for glioma. Trial on the first one is still ongoing and that on the latter is completed but no result is published. While waiting for the results of the new drugs, some existing drugs showed potential to improve radiation effect. Valproic acid was reported to improve radiation injury to meningioma stem-like cells *in vitro*, by elevating the G2/M phase of the cell cycle and inducing cell apoptosis (40). Furthermore, hydroxyurea, which could interfere with DNA repair after radiation, has been reported to improve PFS of AM with incomplete resection, which indicates that this drug is a potent radiosensitizer to radioresistant AM (41).

Our study presents some limitations: the inherent limitation of a retrospective analysis, relatively low number of cases due to the rarity of this kind of tumor, the decision to undergo postoperative ART at the discretion of surgeons rather than objective parameters, and the small number of microRNA samples. However, the present study includes a significant follow-up, and all cases are from a single institution, which avoids the “interinstitutional” diagnostic and therapeutic discrepancies.

In summary, we found 14 differently expressed miRNAs in radiotherapy-sensitive and radiotherapy-resistant AM patients. These miRNAs may be used as candidate predictive markers for the benefit of radiotherapy in AM. Should these results be confirmed in future prospective randomized trials, the miRNA signatures may be used to identify AM patients who may not respond well to adjuvant radiotherapy and may, therefore, benefit from the addition of radiosensitizers or immunotherapy to enhance the radiation response. As such, applying the potential roles of miRNAs in individualized radiotherapy may lead to novel trends in AM therapeutic options.

## Data Availability Statement

The datasets (GENERATED) for this study can be found in the [Gene Expression Omnibus (GEO)] (https://www.ncbi.nlm.nih.gov/geo/query/acc.cgi?acc=GSE144037).

## Ethics Statement

The studies involving human participants were reviewed and approved by Medical Ethics Committee of the Capital Medical University. The patients/participants provided their written informed consent to participate in this study.

## Author Contributions

All authors listed have made a substantial, direct, and intellectual contribution to the work. The idea came from the discussion of SL and YW. Material preparation, data collection, and analysis were performed by XZ, GZ, and HL. The first draft of the manuscript was written by HH and XZ and reviewed by GZ and YW. All authors commented on the manuscript and approved it for publication.

## Conflict of Interest

The authors declare that the research was conducted in the absence of any commercial or financial relationships that could be construed as a potential conflict of interest.
